# The molecular understanding of cancer: from the unspeakable illness to a curable disease

**DOI:** 10.3332/ecancer.2017.747

**Published:** 2017-06-08

**Authors:** Marco A Pierotti

**Affiliations:** FIRC Institute of Molecular Oncology (IFOM), via Adamello, 16 20136, Milan, Italy; Foundation IRP Città della Speranza, Cso Stati Uniti, 4-35127 Padua, Italy

**Keywords:** oncogenic viruses, oncogenes, tumor suppressors, targeted therapy

## Abstract

The beginning of our understanding of the molecular basis of cancer and the discovery in the 1980s of cancer associated genes, oncogenes, and tumour suppressor genes has led to cancer becoming a treatable condition rather than an unspeakable disease. In 1971, the then USA President, Richard Nixon, declared ‘war against cancer’ with a far too optimistic perspective of winning in just a few years. This tactic failed because our knowledge of the disease was still very limited and even its origin—viral or due to exposure to external agents—was still highly debated. A better understanding of the cause(s) of the origin of cancer led to its definition as a genetic disease at the somatic level and heralded a new era for molecular diagnosis and the development of more mechanistic evidence-based, targeted cancer therapies. However, the initial positive results were soon overshadowed by a major limitation of targeted agents, namely resistance mechanisms, which still represent an obstacle for the full eradication of the disease. More recently, effective therapeutic approaches have been developed in the field of ‘immunotherapy’. The combination of novel therapies will hopefully result in effective cancer growth control and make the disease ‘chronic’. The launch of the ‘Moonshot Cancer Program’ by President Barack Obama aims to significantly reduce cancer deaths in the next decade—let us see.

‘I will also ask for an appropriation of an extra 100 millions of dollars to launch an intensive campaign to find a cure for cancer…’.

With this sentence, President Nixon declared the war on cancer in 1971 and in December of the same year he signed the National Cancer Act following through on his promise. Moreover, the timing for this was projected for 1976 to coincide with the Country’s Bicentennial. It was compared to the race to the moon that President Kennedy successfully beat the Russians to a decade earlier. However, this did not happen, what then went wrong? Simply, the conquest of the moon required only investments to put together already existing knowledge and technology. Cancer, on the other hand, was still fundamentally a poorly understood disease. It was still unclear if cancer was due to some external factor, that is, an infectious agent or due to ageing-related pathologies leading to cell degeneration. In fact, at the time of the National Cancer Act signing, two theories on the origin of cancer were debated: the viral hypothesis, evidence for which came mainly from cancer models in rodents, and the environmental hypothesis which, apart from the difficulty in isolating tumour viruses of clear human origin, found its strong evidences from epidemiological observations. The first such observation came in 1775 from a report by Sir Percival Pott in London. This epidemiology pioneer described the first occupational induced cancer: cancer of the scrotum in chimney sweepers chronically exposed to compounds present in chimney dust, subsequently recognised (e.g. benzo (a)pyrene) as potent carcinogens [1]. On the other hand, the concept that a virus could cause cancer found its explanations in the work of another outstanding scientist, Francis Peyton Rous at the Rockefeller University in New York. In 1910 and 1911, he published two works where he reported his seminal observation that a malignant tumour (a spontaneous arisen sarcoma) of a domestic chicken could be transferred to another fowl by injecting the healthy bird with a cell-free filtrate of the original tumour. This procedure followed the so-called Koch’s postulate thus indicating the filtrate contained the sarcoma-causing agent [2, 3].

It took, however, several decades before this discovery was fully appreciated and it was a lucky occurrence that Peyton Rous lived long enough to receive his well-deserved Nobel Prize in 1966. The analysis of the transmissible cell-free agent described by Rous led to the identification of a virus, the Rous sarcoma virus, whose molecular characterisation disclosed basic aspects for the comprehension of the mechanism controlling cell growth and differentiation and therefore, ultimately, cancer. The Rous sarcoma virus had four distinct genes: gag, pol, env, and src, and the latter was eventually recognised as the cancer-causing gene and this led to the definition of ‘oncogene’. In addition, a new enzyme was discovered, encoded by the viral pol gene, the so-called reverse polymerase able to retro-transcribe RNA into DNA, thus violating the central dogma of biology that dictated an unidirectional flux of information starting from DNA to RNA and finally proteins. The reverse polymerase of a retrovirus converts its RNA into DNA which then integrates in the genome of the host cell. Thanks to the work of Peyton Rous and of a number of cancer retrovirologists, in those years, the basis of modern molecular biology was established. As for cancer, several experimental models led to the discovery of a number of oncogenic retroviruses mainly in rodents. In particular, those responsible for mammary tumours in mouse and named MMTV (murine mammary tumour virus) and for sarcomas in rats such as the Harvey rat sarcoma and the Kirsten rat sarcoma viruses both belonging to the so-called RAS oncogene family [4]. What about human cancer and retroviruses? To summarise a long and highly debated story, a direct involvement of a retrovirus in human tumours has not been proven, although a putative human retrovirus was found in some forms of human lymphomas mainly thanks to the work of Robert Gallo [5]. Perhaps these findings were instrumental to suggest to President Nixon that the war against cancer was nearly won, may be through an anticancer viral vaccine. This was not the case but Gallo’s work created the ground to quickly discover the cause of AIDS, the HIV retrovirus. The controversial origin of cancer was finally resolved at the beginning of the 1980s.

The first fundamental discovery was reported in a paper by the group of Michael Bishop and Harold Varmus [6]. They found that the src gene of the Rous sarcoma virus was present as a normal gene in the DNA of all the mammals including human DNA. It was shocking news to realise that what was believed to be a genuine viral cancer gene was indeed part of the normal human genome. For this discovery, in 1986, Bishop and Varmus were awarded with the Nobel Prize. One year later, three papers from the groups of Michael Wigler, Robert Weinberg and Mariano Barbacid concluded the long story on the origin of human cancer. Through a methodology called transfection, by which an exogenous DNA can be introduced in ‘quasi’ normal recipient cells, murine-immortalised fibroblasts, they were able to show that the DNA from a bladder cancer cell line of human origin but not the one from normal cells, was able to cause the complete transformation in a fully growing cancer of the recipient cells. Characterisation of the transforming agent indicated that the latter coincided with the transforming gene originally detected in a rat sarcoma and designated as Hras oncogene [7–9]. Two year later, Barbacid’s laboratory at the National Cancer Institute in Bethesda, in collaboration with our groups at the Istituto Nazionale Tumori in Milan, reported strong experimental evidence that the gene present in the tumour was slightly different from the same gene in normal tissue in the same individual. It was shown, for the first time, that the DNA from a lung adenocarcinoma from a recently surgically treated patient was able to transform the recipient murine fibroblast and that the responsible gene was the Kirsten member of the ras family (Kras). The DNA extracted from normal tissues (lung parenchyma, white blood cells) of the same patient resulted negative and by comparing the sequence of the tumour gene to the normal revealed that they differ at only one letter [10].

One letter enough to trigger cancer. We understood that the enemies are within: some genes in our genome can be altered (mutated) by different factors, including compounds such as those present in chimney dust, radiation, etc. In addition, DNA viruses can also be associated with cancer development. Finally and most importantly, cancers can also develop without any apparent cause, that is, spontaneously or, as in a small percentage of cases, inherited.

The fact that cancer is caused by the alterations in normal genes, especially those regulating cell growth and differentiation processes is now firmly established. To present a simplified picture of the problem, we can recognise two main categories of cancer genes. The first are ‘oncogenes’ coming directly from the oncogenic virus definition and are also known as dominant oncogenes: one altered allele is sufficient to contribute to the malignant phenotype and differ from their normal counterpart for quantitative or timely inappropriate expression or, as for the ras family oncogenes, by structural alterations [11]. It is worth mentioning the Italian contributions to the oncogene field, such as the discovery and characterisation of SHC and PML/RAR alfa by Pier Giuseppe Pelicci’s group [12, 13], MET oncogene in the laboratory of Paolo Comoglio [14] and the thyroid tumour associated oncogenes RET and TRK discovered by a joint collaboration of our group in INT and Giancarlo Vecchio’ s laboratory [15, 16]. A second class of cancer genes was discovered by characterising childhood tumours such as retinoblastoma. Unlike oncogenes, these genes are recessive so both alleles must be lost or inactivated to allow malignant transformation of the cells. These genes were defined as ‘tumour suppressors’ after several experiments showed that their reintroduction into defective tumour cells was able to revert the transformed phenotype. Focusing on their functional properties, we can summarise that while oncogenes can be considered the ‘gas pedal’, tumour suppressor genes act as a brake for cell growth [17]. Transfection experiments using quasi-normal cell lines suggested that mutation of a single gene could cause cancer. This shocking result was later in part mitigated by experiments performed by Weinberg’s group in collaboration with Varda Rotter at Weizmann Institute in Israel, which proved that to fully transform a normal cell at least two different mutations are required: the activation of an oncogene and the inactivation of a tumour suppressor gene [18].

Further details on the pathway leading from normal cells to cancer were identified in Bert Vogelstein’s laboratory that analysed cells isolated from colonic mucosa at different stages of tumour progression. They described at least seven molecular events involving activation of oncogenes and loss of tumour suppressor genes that are necessary to promote a Darwinian selection of a clone possessing all the features of a fast growing and invasive tumour [19].

This model with just minor modifications holds for many tumour types and is in agreement with epidemiological data. We now see cancer, in its widest definition, as a genetic disease at somatic level. Genetic, indeed, is not synonymous for hereditary. The long and interesting story that led to the identification of the familial basis of some cancers cannot be discussed here, but it is worth mentioning that 5–10% of tumours display a clear hereditary pattern.

The genetic basis of cancer inheritance received a molecular explanation when genes such as *Rb* for retinoblastoma, APC for familial polyposis and BRCA 1 and 2 for breast and ovarian cancer were identified and characterised.

All these are examples of ‘tumour suppressing’ genes and cancer formation requires inactivation of both alleles. The peculiarity of hereditary cancers is that one of the two events has already occurred at birth. What is inherited is not actually cancer, but an increased risk of developing cancer [20].

As the history of medicine teaches us we can only start to cure a disease, only once we have clearly understood it. Maybe we are getting closer for cancer. It is now becoming clear that the traditional histopathological, morphologically based classification of tumours could be significantly improved by a complementary molecular characterisation which not only allows a more refined stratification of patients, originally grouped together, but also more specifically correlated with prognostic features (such as therapy response) and ultimately, clinical outcome. An example of this spectacular development is provided by the gene expression analysis of breast cancer cases by microarray, first developed in Charles Perou’s laboratory (at Stanford) in 2000, which revealed that according to gene expression patterns these tumours could be stratified into five distinct subgroups correlating strongly with the patient survival [21].

The molecular characterisation of tumours with the identification of molecular targets necessary for tumour growth and survival, opened a new unprecedented era for a rational approach to cancer therapy, contrasting with the empiric but necessary traditional approach of cytotoxic therapy which worked on tight therapeutic windows only quantitatively discriminating between normal and neoplastic cells. This new approach caused a revolution in the way of thinking about cancer drug therapy both for the pathologists and the medical oncologists. The tumour type, once the reference for a given cytotoxic drug, was no more the issue, but rather the molecular alterations, which drive tumour growth. No longer one drug—one tumour type, but a drug for several tumour types sharing the same molecular alteration. In other words, the targets are now the basis for the therapy and led to the new drug discovery and development field. All this started at the end of the 1990s, with the development by Ciba-Geigy then Novartis and its introduction into the clinic by Brian Druker of Imatinib, originally Gleevec, an ATP antagonist designed to bind to the kinase ABL [22]. The constitutive activation of the enzyme following a chromosomal translocation juxtaposing two loci ABL and BCR normally located on two different chromosomes was recognised as the growth driving and survival mechanism for chronic myelogenous leukemia (CML). All CML cells display the so-called Philadelphia chromosome which is the structural consequence of a t(9;22) chromosomal translocation leading to the production of a tumour-specific fusion protein in which the active enzymatic component, ABL kinase, resulted necessary for tumour survival. By switching off the enzyme the tumour cells stopped to grow and died. The long waited cancer cure seemed to be accomplished. However, CML has unique properties: it is a highly homogeneous tumour with all the cells carrying the same BCR/ABL molecular defect and the tumour cells are addicted to the activity of this oncogene. Although many molecular targets have been described in many tumour types and the relative potential targeted therapies have been since then developed, two correlated problems have been experienced in this potentially effective therapeutic approach. First, cancer heterogeneity, which characterises the majority of solid tumours also at the molecular level. Second, the understanding that, as also in the case of imatinib and CML, this kind of therapy does not completely eradicate cancer cells but instead exerts only a cytostatic activity. This means these kinds of drug must be provided chronically otherwise the patient will rapidly relapse. In addition, even though the drug is always present, the tumour often reappears with some new molecular properties rendering the cells resistant to the original drug. Fortunately, through a combination of molecular analysis, molecular modelling and medicinal chemistry, it is possible to develop variants of the original drug for second- and even third-line treatments.

These findings tell us that even with these new promising perspectives we are still in the middle of our battle against cancer [23]. In the most recent years, new weapons have been developed. These new therapeutic opportunities came from an old concept of the 1960s ‘the Immunosurveillance theory of cancer’ proposed originally by Frank Mcfarlene Burnet [24]. According to his theory, we develop every day a cancer cell that is (often) recognised and destroyed by our immune system. When things go wrong, a cancer develops. So the idea of immunotherapy was developed. For this, we need to better understand how the immune system recognises and then destroys cancer cells and why in the majority of tumours this does not happen. This was an unsolved issue in cancer immunology for a long time. Experimental evidences from the relationship between inflammation and cancer provided a first insight into the problem. As in other pathological conditions, an inflammatory response is useful to the organism but if not controlled it could be detrimental. In cancer, both a beneficial inflammatory anticancer response but also a tumour-promoting inflammatory activity have been demonstrated [25, 26]. A more accurate analysis and knowledge of the mechanisms underlying this relationship will open up new therapeutic approaches as potential targets for therapy and not only for the tumour cell itself but also the surrounding environment.

In the cancer immunology field, however, the real breakthrough was the introduction into the clinic of the first effective and rationale immunotherapy procedures. In 2010, in fact, *The New England Journal of Medicine* reported the up until then unbelievable positive results of a multicentric clinical trial in metastatic melanoma, one of the most deadly and untreatable cancers, using a humanised monoclonal antibody, ipilimumab [27]. This therapeutic agent was developed thanks to the pioneering studies of James Allison who showed that in principle an antitumour T cell, response can be triggered by the organism. However, due to the induction of modulator molecules such as PD-1 and the cognate ligand and other components of this feedback machinery, called checkpoint molecules, such as CTLA4, soon this response is blocked [28]. Therefore, they developed the therapeutic concept of inhibiting the blockers to unlock the response using humanised monoclonal antibodies, such as ipilimumab against the relevant checkpoint molecules [29].

Although we still have to carefully evaluate the side effects of this approach and still do not understand why in some individuals it does not work, certainly more research and the development of strategies employing, for example, radiotherapy to improve the ‘immunogenicity’ of the tumour before the immunotherapy and/or using combinations of targeted therapy and immunotherapy, give us reasons for an optimistic view to control cancer growth.

The former USA Administration shared this view since President Obama appointed Vice President Joe Biden to lead the so-called Moonshot Cancer Project to promote and financially support these new efforts to defeat cancer. Hopefully, this presidential willingness will have a different outcome to President Nixon’s efforts because, as we have discussed before, the nature of cancer is no more a black box and considerable progress has been made in the past decade in reducing its mortality rate. The time is right to dream as Umberto Veronesi often declared: to have a world without deaths from cancer.
